# Isolated posterior mitral leaflet elongation as a rare mechanism of systolic anterior motion in hypertrophic cardiomyopathy: a case report of successful treatment with mavacamten

**DOI:** 10.1093/ehjcr/ytag528

**Published:** 2026-07-11

**Authors:** Luis Enrique Gomez, Paula Inés Buonfiglio, Judith Ackerman, Carlos David Bruque, Andres Nicolas Atamañuk

**Affiliations:** Inherited Heart Disease Clinic, Hospital General de Agudos “Dr. Juan A. Fernández”, Cerviño 3356, C1425AGP Buenos Aires, Argentina; Inherited Heart Disease Clinic, Hospital General de Agudos “Dr. Juan A. Fernández”, Cerviño 3356, C1425AGP Buenos Aires, Argentina; Pediatric Cardiology, Hospital General de Niños “Dr. Pedro de Elizalde”, Av. Montes de Oca 40, C1270AAN Buenos Aires, Argentina; Unidad de Conocimiento Traslacional Hospitalaria Patagónica, Hospital SAMIC El Calafate, Jorge Newbery 453, Z9405CBC El Calafate, Santa Cruz, Argentina; Inherited Heart Disease Clinic, Hospital General de Agudos “Dr. Juan A. Fernández”, Cerviño 3356, C1425AGP Buenos Aires, Argentina

**Keywords:** Hypertrophic cardiomyopathy, Systolic anterior motion, Posterior mitral leaflet, Mavacamten, Genetic testing, Case report

## Abstract

**Background:**

Hypertrophic cardiomyopathy (HCM) is the most common inherited cardiac disorder. Left ventricular outflow tract (LVOT) obstruction typically results from systolic anterior motion (SAM) of the anterior mitral leaflet.

**Case summary:**

We describe a 26-year-old man with HCM in whom SAM was generated by isolated elongation of the posterior mitral leaflet, a rare mechanism with important clinical implications. He was diagnosed during childhood by a paediatric cardiologist and followed until transition to adult care. On presentation, he was in NYHA (New York Heart Association) class II with asymmetric septal hypertrophy (25 mm) and posterior leaflet elongation causing LVOT obstruction. Cardiac magnetic resonance confirmed hypertrophy and late gadolinium enhancement at the site of maximal wall thickness. Genetic testing revealed a pathogenic *MYH7* variant (p.Ile263Thr) and a *MYH6* classified as of uncertain significance. Treatment with mavacamten 5 mg led to a reduction of the LVOT gradient from 63 to 32 mmHg and symptomatic improvement.

**Discussion:**

This case illustrates an unusual anatomical substrate for LVOT obstruction in HCM and emphasizes the role of multimodality imaging in diagnosis. It also highlights the therapeutic potential of myosin inhibition with mavacamten in avoiding invasive interventions in complex phenotypes.

Learning points

- Multimodality imaging is essential for detecting atypical mechanisms of SAM.
- Novel targeted pharmacologic therapy can substantially alter management strategies in complex HCM phenotypes.

## Introduction

Hypertrophic cardiomyopathy (HCM) is the most frequent inherited cardiac disorder, with an estimated prevalence of 1 in 500 individuals.^1,2^ Approximately half of cases are inherited in an autosomal dominant manner, conferring a 50% transmission risk^3^ Diagnosis is established by a left ventricular (LV) wall thickness ≥15 mm, supported by family history, genetic testing, or ECG features in borderline cases.^4^

Genetic testing identifies a pathogenic variant in 40–60% of patients, particularly in familial and early-onset forms.^3,5,6^ Most pathogenic variants affect genes that encode sarcomeric proteins, with the most commonly implicated being *MYH7* (beta-myosin heavy chain 7) and *MYBPC3* (myosin-binding protein C3).^7^

From a pathophysiological perspective, systolic anterior motion (SAM) of the mitral valve is a hallmark mechanism of left ventricular outflow tract (LVOT) obstruction.^8^ This typically involves elongation of the anterior mitral leaflet; posterior leaflet involvement is rarely described.

## Summary figure

**Table:** 

Timepoint	Event
Childhood	Diagnosis of HCM by paediatric cardiologist
Adolescence–early adulthood	Regular follow-up at paediatric hospital
Transition	Transferred to adult cardiology care
Presentation (26 years)	NYHA II; asymmetric septal hypertrophy; SAM due to posterior leaflet elongation; LVOT obstruction (63 mmHg)
Cardiac MRI	Confirmed septal hypertrophy; LGE at maximal wall thickness; posterior leaflet elongation
Genetic testing	Pathogenic MYH7 (p.Ile263Thr); MYH6 VUS (p.Arg1691His)
Treatment	Mavacamten 5 mg initiated
4 weeks follow-up	LVOT gradient reduced to 32 mmHg; symptomatic improvement; preserved LV systolic function

## Case presentation

### Medical and family history

The proband was a 26-year-old man referred for evaluation of suspected HCM. He had been diagnosed in childhood by his paediatric cardiologist and was followed longitudinally at a paediatric hospital until transition to adult care. At presentation to our centre, he was in NYHA (New York Heart Association) functional class II. He had no cardiovascular risk factors and denied chest pain, syncope, or palpitations. Family history was negative for cardiomyopathy, sudden cardiac death, or premature coronary disease.

### Physical examination

Blood pressure was 100/60 mmHg, and heart rate was 50 b.p.m. Cardiac auscultation revealed a systolic ejection murmur best heard at the aortic base, which increased in intensity with the Valsalva manoeuvre. No signs of heart failure were present.

### Electrocardiography

Twelve-lead ECG demonstrated sinus rhythm, left bundle branch block (LBBB), and T-wave inversions in leads I, aVL, V1, and V2.

### Echocardiography

Transthoracic echocardiography revealed asymmetric septal hypertrophy with a maximal septal thickness of 25 mm. Left ventricular systolic function was preserved. A mild anterior mitral leaflet prolapse without significant regurgitation was observed. Importantly, there was isolated elongation of the posterior mitral leaflet, measuring 22 mm, which protruded through the inter-chordal space and contacted the interventricular septum, generating SAM and dynamic LVOT obstruction (*Figure 1*).

**Figure 1:**
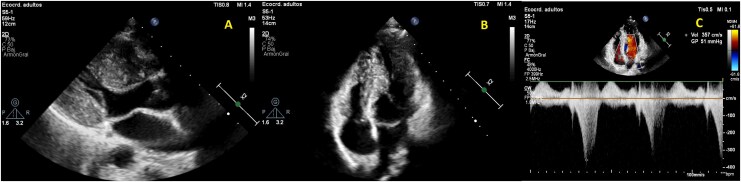
Echocardiogram: (*A*) parasternal long-axis view and (*B*) paraesternal four-chamber view. Isolated elongation of the posterior leaflet (measuring 22 mm) causes systolic anterior motion, the residual leaflet protrudes through the spaces between the chordae to contact the septum. Although an uncommon cause of mitral-septal contact, it nonetheless can cause severe obstruction. (*C*) A late-peaking systolic gradient with peak pressure gradient of 51 mmHg at rest.

### Cardiac magnetic resonance

Cardiac MRI confirmed the echocardiographic findings of asymmetric septal hypertrophy consistent with HCM. Left ventricular systolic function was normal. Late gadolinium enhancement (LGE) was present at the site of maximal wall thickness, consistent with focal replacement fibrosis. Importantly, MRI also corroborated the presence of posterior mitral leaflet elongation contributing to SAM and dynamic LVOT obstruction, in agreement with echocardiographic observations (*Figure 2*).

**Figure 2:**
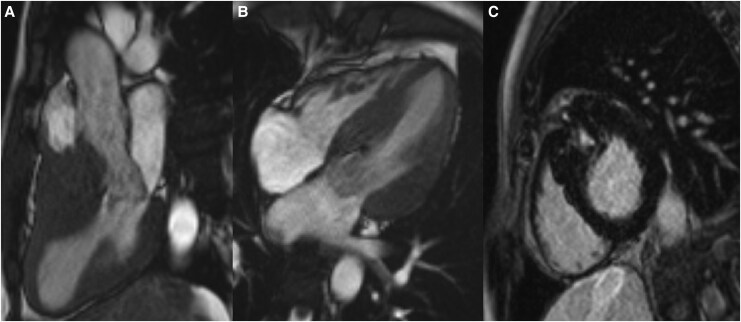
Cardiac MRI: (*A*) parasternal long-axis view and (*B*) paraesternal four-chamber view, confirming the presence of posterior mitral leaflet elongation contributing to systolic anterior motion and dynamic left ventricular outflow tract obstruction. (*C*) Late gadolinium enhancement was present at the site of maximal wall thickness.

### Clinical course

The patient had previously been treated with atenolol (50 mg twice daily), prescribed at a paediatric cardiology clinic. Further optimization of first-line pharmacological therapy was limited by baseline sinus bradycardia (∼50 b.p.m.) and relative hypotension (100/60 mmHg), precluding safe up-titration of β-blockers and the use of non-dihydropyridine calcium channel blockers; disopyramide was also considered unsuitable, primarily because it is not commercially available in our country. Mavacamten was subsequently initiated on an individualized basis, given the haemodynamic limitations of conventional therapy and in line with emerging evidence supporting myosin inhibition in obstructive HCM.

### Genetic findings

Next-generation sequencing identified two variants: *MYH7* (NM_000257.4): c.788T > C (p.Ile263Thr) and *MYH6* (NM_002471.3): c.5072G > A (p.Arg1691His). According to ACMG/ClinVar criteria,^9^ the MYH7 variant is classified as pathogenic, consistent with its established role in sarcomeric hypertrophic cardiomyopathy and supporting the genetic diagnosis in this patient. In contrast, the MYH6 variant is currently classified as a variant of uncertain significance, as its association to HCM remains less well defined and it has been more commonly associated with congenital heart disease than with classic sarcomeric HCM.

### Treatment and follow-up

Given the presence of symptomatic LVOT obstruction and the unusual mechanism of SAM, the patient was started on mavacamten 5 mg daily. Within 4 weeks, a marked reduction in the LVOT gradient was documented, decreasing to 32 mmHg, accompanied by clinical improvement. Post-treatment LVOT gradient was assessed using resting echocardiography and provocative manoeuvres (Valsalva), as exercise echocardiography had not been performed. Importantly, pre-treatment evaluation was conducted under the same conditions, allowing for consistent comparison of LVOT gradients. Follow-up echocardiography showed reduction of flow acceleration and attenuation of SAM dynamics, while left ventricular systolic function remained preserved (*Figure 3*).

**Figure 3:**
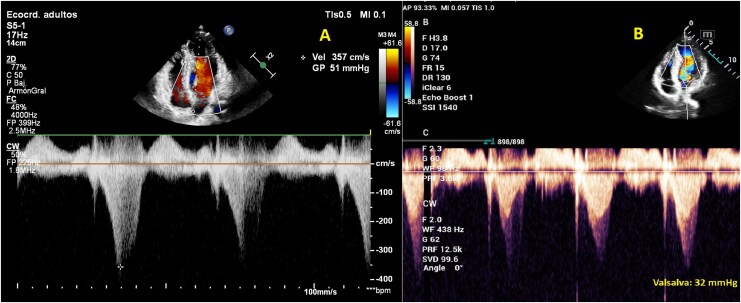
(*A*) Baseline echo showing posterior leaflet elongation with systolic anterior motion and left ventricular outflow tract obstruction (63 mmHg). (*B*) After 4 weeks of mavacamten 5 mg, left ventricular outflow tract gradient reduced to 32 mmHg with attenuation of systolic anterior motion.

## Discussion

This case highlights an unusual mechanism of LVOT obstruction due to posterior mitral leaflet elongation in the context of HCM. While SAM typically involves the anterior leaflet, posterior leaflet elongation can also produce significant obstruction.

From a management perspective, recognition of this anatomical variant is critical for surgical planning, as mitral valve morphology influences the choice of intervention. The posterior mitral leaflet showed marked elongation (22 mm), clearly exceeding the expected normal range (∼11–14 mm),^10^ which may have implications for surgical strategies, including consideration of leaflet plication.^11^ In this case, the decision to initiate mavacamten was driven by haemodynamic limitations to further optimization of first-line therapy, including baseline bradycardia and relative hypotension. This highlights the importance of individualized treatment strategies in obstructive HCM, particularly when conventional pharmacological options are not well tolerated. In this context, emerging evidence supports the use of myosin inhibitors such as mavacamten, as demonstrated in trials including EXPLORER-HCM and VALOR-HCM.^12–14^ In our patient, the introduction of mavacamten, a cardiac myosin inhibitor, effectively reduced the LVOT gradient and alleviated symptoms, obviating the immediate need for septal reduction therapy. However, given the structural contribution of PMVL elongation to LVOT obstruction, long-term follow-up will be important to assess the durability of response to mavacamten and the potential future need for mitral valve intervention.

This case therefore illustrates two teaching points: (i) multimodality imaging is essential for detecting atypical mechanisms of SAM and (ii) novel targeted pharmacologic therapy can substantially alter management strategies in complex HCM phenotypes.

## Conclusions

We describe a young patient with HCM and LVOT obstruction caused by posterior mitral leaflet elongation. Multimodality imaging clarified the mechanism, and treatment with mavacamten resulted in significant haemodynamic and symptomatic improvement. This case expands the anatomical spectrum of obstruction in HCM and demonstrates the therapeutic potential of myosin inhibition in individualized care.

## Data Availability

Data sharing is not applicable to this article as no new data were created or analysed in this study.

## Lead author biography

Dr Luis Enrique Gomez earned his medical degree from the University of Buenos Aires in 2005 and trained in Cardiology at the Italian Hospital of Buenos Aires. He later specialized in congenital heart disease and, in 2020, he founded the Hereditary Cardiovascular Diseases Clinic at Hospital Fernández while pursuing a Master’s in Genetics. He currently leads Adult Congenital Heart Disease Programs at Hospital Fernández and the Italian Hospital. A faculty member at the Argentine Society of Cardiology since 2013, he is deeply engaged in teaching, mentoring, and research, and will preside over SAC’s Genetic Cardiology Council in 2025.

## Notes

**Consent:** Written informed consent was obtained from the patient for publication of this case report and any accompanying images, in accordance with COPE guidelines.
